# Dietary Amylose/Amylopectin Ratio Modulated Growth Performance, Intestinal Health, and Lipid Metabolism in Goslings Fed Low-Protein Diets

**DOI:** 10.3390/ani16020189

**Published:** 2026-01-08

**Authors:** Xiyuan Xing, Xucheng Zheng, Xuan Li, Zhi Yang, Haiming Yang, Zhiyue Wang

**Affiliations:** 1 College of Animal Science and Technology, Yangzhou University, Yangzhou 225009, China; mz120231558@stu.yzu.edu.cn (X.X.); dx120230175@stu.yzu.edu.cn (X.Z.); liooo1111@163.com (X.L.); hmyang@yzu.edu.cn (H.Y.); 2 Joint International Research Laboratory of Agriculture and Agri-Product Safety, Ministry of Education of China, Yangzhou University, Yangzhou 225009, China; zhiyang@yzu.edu.cn

**Keywords:** low-protein diet, amylose/amylopectin ratio, growth performance, lipid metabolism, intestinal health, meat quality, goslings

## Abstract

This study investigated the effects of amylose/amylopectin (AM/AP) ratios in low-protein (LP) diets on the growth performance, fat deposition, and nutrient utilization in goslings. A total of 288 healthy, 35-day-old male Jiangnan White Geese were randomly divided into four treatment groups: one group fed a normal protein diet (16%) with an AM/AP ratio of 0.34 (NPR_0.34_), and three groups fed low protein diets (14%) with different AM/AP ratios (LPR_0.26_, LPR_0.34_, LPR_0.44_). Each group consisted of six replicates, with 12 geese per replicate, and they were fed for 28 days. The results showed that the body weight at 63 days and average daily gain (ADG) of the LPR_0.44_ group geese were significantly higher than those of the other groups (*p* < 0.01), while the feed-to-gain ratio (F/G) was lower (*p* < 0.05). The abdominal and mesenteric fat contents were lower in the LPR_0.44_ group than in the LPR_0.26_ group (*p* < 0.05), whereas the breast and leg muscle yields were higher (*p* < 0.05). The breast muscle redness (a*) of the LPR_0.34_ and LPR_0.44_ groups was higher than in the NPR_0.34_ group at 45 min (*p* < 0.05). The levels of C6:0, C8:0, C11:0, C12:0, and C13:0 in breast muscle saturated fatty acids (SFAs) of the LPR_0.44_ group were higher, while that of C18:0 was lower compared with the LPR_0.26_ group (*p* < 0.05). The serum total cholesterol (TC) and triglycerides (TGs) in the LPR_0.44_ group were lower than in the LPR_0.26_ group (*p* < 0.05). Hepatic lipase (HL) activity was significantly lower in the LPR_0.44_ group (*p* < 0.01). Regarding hepatic fatty acids, the levels of butyric acid (C4:0), lauric acid (C12:0), and nervonic acid (C24:1) were lower in the LPR_0.44_ group than in the LPR_0.26_ group (*p* < 0.05). No significant differences were observed in intestinal morphology, digestive enzyme activities, or nutrient utilization among the groups. (*p* > 0.05). In conclusion, adjusting the AM/AP ratio to 0.44 in a low-protein diet improved growth performance, regulated lipid metabolism, and maintained intestinal function in goslings.

## Simple Summary

This study investigated the effects of adjusting the amylose to amylopectin (AM/AP) ratio in a low-protein (14%) diet on growth performance, lipid metabolism, and intestinal health in goslings. The results demonstrate that an AM/AP ratio of 0.44 significantly enhanced average daily gain and feed efficiency, reduced abdominal and mesenteric fat deposition, and improved breast muscle yield and meat redness, without adversely affecting nutrient digestibility or intestinal morphology. These findings propose an innovative nutritional strategy to mitigate the excessive fat deposition commonly induced by low-protein diets in poultry. Furthermore, by enabling the efficient use of low-protein diets while controlling fat accumulation, this study contributes to sustainable poultry production and enhanced goose farming efficiency.

## 1. Introduction

The limited availability of protein and energy feed resources has become a critical constraint on the sustainable development of the Chinese livestock industry. This challenge is particularly pronounced in the current transition of animal husbandry toward precision nutrition and environmentally sustainable (green) farming practices. In recent years, low-protein diets have demonstrated considerable potential in animal production systems as an effective strategy to reduce nitrogen emissions and feeding costs. Reducing dietary crude protein from 16% to 13% has been shown to maintain laying hen performance and feed efficiency while decreasing nitrogen emissions by more than 40% [1,2]. A moderate 2–3% reduction in dietary protein has been shown to not compromise performance while significantly improving protein utilization efficiency in poultry production [3,4]. However, dietary protein reduction influences lipid metabolism. Studies have demonstrated that protein-restricted diets require a higher inclusion of cereal grains (e.g., corn, wheat) [5], and this increase in dietary starch may promote excessive energy storage in the form of abdominal fat [6]. Furthermore, Low-protein feeds have been shown to activate lipogenic pathways, significantly elevating serum and hepatic triglyceride (TG) concentrations and body fat content in laying hens [7]. Excessive fat accumulation presents multiple detrimental effects, including reduced feed conversion efficiency, increased production costs, impaired organ function, and compromised carcass quality. More critically, it may predispose poultry to metabolic disorders such as fatty liver syndrome, posing substantial health risks [8]. Therefore, developing nutritional strategies to mitigate excessive fat deposition induced by low-protein diets is essential to ensure poultry health and enhance production efficiency.

Starch serves as the primary carbohydrate source in poultry diets, and its digestive characteristics directly determine energy utilization efficiency [9]. Starch consists of two polysaccharides, amylose and amylopectin; when the ratio of AM/AP in starch is relatively high, its linear molecules are more likely to form a dense helical structure and an enzyme-resistant gel network through hydrogen bonds, thereby slowing down the digestion rate and transforming starch from “rapidly digestible” to “slowly digestible” and even “resistant” types [10]. Because a high AM/AP ratio has strong resistance to digestion and is relatively slowly digested and absorbed in the intestine, it can continuously provide energy, which is beneficial to the growth and development of animals [11]. The ratio between them (AM/AP) plays a crucial role in modulating animal lipid metabolism [12]. A high AM/AP ratio can reduce the digestion and utilization of starch in the intestine, regulate blood sugar by slowly releasing glucose, inhibit fatty acid synthase activity [11], and ultimately reduce the synthesis of liver and serum triglycerides (TGs), effectively preventing excessive body fat deposition [9]. Studies demonstrate that amylose regulated blood glucose through controlled release, inhibits fatty acid synthase activity to reduce fat deposition and significantly decreases adipocyte size and body weight gain in rodent models [11,13]. In broiler production, slowly digestible starch effectively reduced abdominal fat deposition by modulating serum triglyceride concentrations [14]. Collectively, these findings demonstrated that increasing the AM/AP ratio in feeds benefits both animal growth performance and lipid metabolism regulation.

In conclusion, against the backdrop of resource constraints and environmental protection transformation, low-protein diets are becoming important feeding strategies owing to their advantages such as saving protein resources and reducing nitrogen emissions. However, simply reducing protein levels can easily lead to lipid metabolism disorders and excessive body fat deposition. This is because reducing dietary protein levels often requires increasing the proportion of grains to maintain energy supply, resulting in increased starch intake, and excess carbohydrate energy is easily converted into fat storage. Starch is the main carbohydrate source, and its amylose to amylopectin ratio (AM/AP) is considered to be a key factor in regulating glucose release and lipid metabolism. Current research on goose nutrition mainly focuses on the conventional protein level (16–18%) [15,16], whereas research on regulating lipid metabolism by adjusting the ratio of AM/AP under the condition of low protein (14%) is still relatively lacking. In addition, previous studies have confirmed that low-protein diets supplemented only with L-lysine and DL-methionine without the addition of extra synthetic amino acids have no impact on the production performance of geese [17], and another study also shows that supplementing only L-lysine and DL-methionine in low-protein diets will not have obvious impacts on the production performance of goslings from 1 to 28 days old [5]. Based on the above reasons, this study aimed to explore the effects of adjusting the AM/AP ratio in a 14% low-protein diet (only supplemented with L-lysine and DL-methionine) on the growth performance, lipid metabolism and nutritional metabolism efficiency of goose chicks, thereby providing a scientific basis for precise nutrition in the goose production system.

## 2. Materials and Methods

### 2.1. Ethics Statement

These experimental procedures were approved by the Institutional Animal Care and Use Committee of Yangzhou University (Approval No.202403131). As the animal sampling procedures were covered by being pre-approved for commercial implementation, no additional license was required. Humane techniques, including rapid cervical dislocation, were used throughout the study and were conducted with consistent adherence to minimize animal discomfort, in strict compliance with animal welfare and ethical standards.

### 2.2. Experimental Design and Feed Formulation

A total of 288 healthy, 35-day-old Jiangnan White geese of similar weight (2122.72 ± 5.16 g) were purchased from Changzhou Siji Poultry Co., Ltd. (Changzhou, China). A single-factor randomized design was employed, with geese randomly assigned to four treatment groups, each consisting of six replicates with 12 geese per replicate. The NPR_0.34_ group received a basal diet (corn-soybean meal diet), while experimental groups were fed low-protein (14%) diets with different AM/AP ratios: 0.26 (LPR_0.26_), 0.34 (LPR_0.34_), and 0.44 (LPR_0.44_). High-amylose corn starch (amylose content = 74.6%, amylopectin content = 25.4%) and high-amylopectin corn starch (amylopectin content > 99%) were used to adjust the AM/AP ratio in the diets. Both starch sources were provided by Shanghai Quanwang Biotechnology Co., Ltd. (Shanghai, China). All treatment groups received diets with a metabolizable energy level of 11.20 MJ/kg. The present experiment was conducted at Yangzhou University’s Gaoyou Experimental Farm (Yangzhou, China), from August to November 2024. The ingredient composition and nutrient levels of the diets are shown in Table 1. The main raw material ingredients are as follows: corn (crude protein: 8.0%, starch: 63.5%), soybean meal (crude protein: 43%, starch: 3.5%), bran (crude protein: 14.33%, starch: 19.8%), and rice husk (crude protein: 2.6%, starch: negligible).

### 2.3. Growth Performance

The body weight (BW) of the goose and the feed intake of each repetition were recorded every week, and the average daily feed intake (ADFI), average daily gain (ADG) and feed-to-gain ratio (F/G) were calculated.

### 2.4. Slaughter Performance

From each replicate, one bird with a body weight closest to the group average was selected. After its live weight was measured, the bird was slaughtered. The carcass traits were measured according to published procedures [18]. The carcass yield, eviscerated yield, semi-eviscerated yield, breast muscle yield, leg muscle yield, abdominal fat yield, skin and subcutaneous fat yield and mesenteric fat yield were determined by the following formulas:

$$carcass\;yield\left(\%\right)=\frac{carcass\;weight}{live\;body\;weight}\times 100\%$$

$$semi-eviscerated\;carcass\;yield\left(\%\right)=\frac{semi-eviscerated\;carcass\;weight}{live\;body\;weight}\times 100\%$$

$$eviscerated\;carcass\;yield\left(\%\right)=\frac{eviscerated\;carcass\;weight}{live\;body\;weight}\times 100\%$$

$$breast\;muscle\;yield\left(\%\right)=\frac{breast\;muscle\;weight}{eviscerated\;carcass\;yield}\times 100\%$$

$$leg\;muscle\;yield\left(\%\right)=\frac{leg\;muscle\;weight}{eviscerated\;carcass\;yield}\times 100\%$$

$$abdominal\;fat\;yield\left(\%\right)=\frac{abdominal\;fat\;weight}{eviscerated\;carcass\;yield+abdominal\;fat\;weight}\times 100\%$$

$$skin\;and\;subcutaneous\;fat\;yield\left(\%\right)=\frac{\left(skin\;and\;subcutaneous\;fat\;weight+abdominal\;fat\;weight\right)}{\left(eviscerated\;carcass\;yield+abdominal\;fat\;weight\right)}\times 100\%$$

$$mesenteric\;fat\;yield\left(\%\right)=\frac{mesenteric\;fat\;weight}{\left(eviscerated\;carcass\;yield+mesenteric\;fat\;weight\right)}\times 100\%$$

### 2.5. Meat Quality

At 63 days of age, after slaughter, the breast muscle of each goose was completely collected for the determination of meat quality indices.

#### 2.5.1. Physical Quality Traits

A portion of the breast muscle was carefully trimmed to remove visible fat, connective tissues, and blood vessels, then standardized into cubes (1 × 1 × 3 cm, 3 cm^3^). After initial weighing, the samples were individually suspended on thin cotton strings in a refrigerated chamber at 4 °C for 24 h, avoiding contact between samples and with the chamber walls. They were then reweighed to calculate drip loss as the percentage of weight reduction.

The pH was measured at 45 min and 24 h post-slaughter using a pH meter (pH-STAR Carcass Meat Quality, Beijing Pengyang Fengye Technology Co., Ltd., Beijing, China). For pH determination, the probe was fully inserted into the muscle at three random sites, and the mean value was used to ensure accuracy.

The color of the breast muscle was measured at 45 min post-slaughter using a colorimeter (CR-400 colorimeter, Konica Minolta Sensing, Inc., Osaka, Japan). For color analysis, triplicate measurements of brightness (L*), redness (a*), and yellowness (b*) were obtained using the colorimeter, and mean values were calculated for statistical analysis.

The meat samples were immersed in a 72.0 °C water bath (ensuring full submersion and avoiding contact between the bag opening and water). The core temperature was continuously monitored until it reached 70 °C, at which point the samples were immediately cooled under running water for 30 min to prevent water infiltration. Cooking loss was calculated as the percentage of weight lost after cooking relative to the initial sample weight.

After cooling, samples were re-equilibrated at room temperature for 30 min. Surface moisture was blotted dry using absorbent paper before cutting along the muscle fiber orientation into 1 cm × 1 cm × 4 cm strips. Visible connective tissues and blood vessels were carefully removed, with a minimum of three strips prepared per sample. Shear force measurement was performed perpendicular to the muscle fiber direction using a meat tenderness tester (RH-N50, Guangzhou Runhu Instrument Technology Co., Ltd., Guangzhou, China), and the mean values were recorded for analysis.

#### 2.5.2. Routine Nutrients

The moisture, crude protein, and crude fat contents in breast muscle and liver were determined according to GB/T 14924.9-2001 [19].

#### 2.5.3. Fatty Acids

For the analysis of 37 fatty acids according to published procedures [20], 0.25 g of muscle or liver tissue samples was weighed. After chloroform/methanol/BHT homogenization and overnight incubation at 4 °C, the samples were sequentially subjected to filtration, calcium chloride centrifugation, and drying. The filtrate was then concentrated by nitrogen blowing, spiked with a known amount of triglyceride (C11:0 TAG) as an internal standard, followed by toluene internal standard derivatization, boron trifluoride/methanol methyl esterification, and n-hexane extraction. The final solution was filtered, evaporated under nitrogen, and reconstituted in n-hexane for gas chromatography analysis (Agilent 7697A, Agilent Technologies, Inc., Santa Clara, CA, USA). The concentration of individual fatty acids was quantified based on peak area and expressed as a percentage of total fatty acids [21].

The samples were analyzed based on the retention times, peak areas, and known concentrations of a 37-component fatty acid methyl ester (FAME) mix standard. The contents of the 37 FAMEs were quantified and then converted to the corresponding fatty acid contents using a standard formula, with reference to the calculation method specified in GB 5009.168-2016 [22].

### 2.6. Serum Metabolites

Blood samples were collected from the wing veins of goslings in a non-fasting state (without feed deprivation) and centrifuged at 3500 rpm for 10 min at 4 °C using a Cence DL-5M low-speed refrigerated centrifuge to obtain serum, which was stored at −20 °C until analysis. Serum concentrations of triglycerides (TG), total cholesterol (TC), total protein (TP), albumin (ALB), globulin (GLB), glucose (GLU), uric acid (UA), high-density lipoprotein (HDL), low-density lipoprotein (LDL), creatinine (CREA), and urea nitrogen (UREA) were measured using an automated biochemical analyzer (UniCel DxC 800 Synchron, Beckman Coulter, Brea, CA, USA).

### 2.7. Liver Lipid Metabolism-Related Enzymes

Liver samples were collected and stored at −80 °C. The activities of lipid metabolism-related enzymes, including fatty acid synthase (FAS), acetyl-CoA carboxylase (ACC), lipoprotein lipase (LPL), and hepatic lipase (HL), were measured by ELISA. The ELISA kits for FAS (Cat. No.: YD-060119G), ACC (Cat. No.: YD-010303G), LPL (Cat. No.: YD-121612G), and HL (Cat. No.: YD-081200G) were purchased from Shanghai Yuduo Biotechnology company (Shanghai, China) and used following the manufacturer’s instructions.

### 2.8. Intestinal Digesta Enzyme

After the geese were slaughtered, intestines were removed, and the duodenal, jejunal, and ileal chyme were immediately collected, placed in cryotubes, flash-frozen in liquid nitrogen, and stored at −80 °C. The activities of α-amylase, lipase, chymotrypsin, and trypsin were measured using kits provided by Nanjing Jiancheng Bioengineering Institute (Nanjing, China). The kits for α-amylase, lipase, chymotrypsin, and trypsin were designated as C016-1-1, A054-2-1, A080-3-1, and A080-2, respectively.

### 2.9. Intestinal Morphometric Assessment

Approximately 1 cm tissue segments of duodenum, jejunum, and ileum were collected and fixed in 4% paraformaldehyde, with fixative replaced after 24 h. Following fixation, tissues were processed through standard paraffin embedding protocols, sectioned at 5 μm thickness, and stained with hematoxylin and eosin (H&E) [23]. Histological sections were examined under light microscopy, with morphometric measurements including villus height (from villus tip to villus-crypt junction), crypt depth (from villus-crypt junction to crypt base), and muscularis propria thickness (vertical distance from mucosal epithelium to muscularis mucosae) performed using a calibrated video imaging system (LY-WN-HPSUPER CCD). For each goose, three complete villus-crypt units were measured per intestinal segment, and the mean value was used for statistical analysis.

### 2.10. Apparent Nutrient Digestibility

During the last week of the experiment, one bird with a body weight closest to the group average was selected from each replicate group. After a 3-day ad libitum pre-test, the formal collection experiment was conducted. Feces were collected for 3 days using the total collection method, with 10% hydrochloric acid added at a ratio of 10 mL per 100 g of feces for nitrogen fixation. The collected fecal samples and test feeds were dried to constant weight at 65 °C, then air-equilibrated for 24 h, ground through a 40-mesh sieve, and stored at −20 °C until analysis. The gross energy (GE), crude protein (CP), crude fat (EE), crude ash, calcium, phosphorus, crude fiber, and dry matter (DM) contents of the feed and fecal samples were analyzed according to the procedures specified by the Official Methods of Analysis [24]. Crude protein was determined using the Kjeldahl method with a Kjeltec system (FOSS NIRSystems Inc., Hillerød, Denmark). Crude fat was measured using a FoodALYTRD40 fat analyzer (FoodALYT, Cologne, Germany), and crude fiber was determined using an Alva automatic fiber analyzer F1600 (Alva Instruments Co., Ltd., Hangzhou, China). Energy was measured using an automatic calorimeter HYLRY-6000 (Changsha Huayuan Chemical Technology Co., Ltd., Changsha, China) [18]. The apparent digestibility of nutrients was calculated as follows:

$$apparent\;digestibility\left(\%\right)=\frac{\left(daily\;intake\;of\;a\;nutrient\;in\;diet-daily\;excretion\;of\;the\;nutrient\;in\;feces\right)}{daily\;intake\;of\;the\;nutrient\;in\;diet}\times 100\%$$

### 2.11. Statistical Analysis

One-way ANOVA was performed for between-group comparisons, and Tukey’s test was applied for post hoc multiple comparisons. All statistical analyses were performed using SPSS 26.0 software (SPSS Inc., Chicago, IL, USA). Differences were considered statistically significant when *p* < 0.05, and data were presented as means and standard error of the mean (SEM).

## 3. Results

### 3.1. Growth Performance

Table 2 shows the effects of low-protein diets with different AM/AP ratios on growth performance in goslings aged 35–63 days. At low protein levels, BW and ADG of goslings increased with increasing AM/AP ratios in the diet. Compared with the LPR_0.26_ group, the LPR_0.44_ group significantly improved the growth performance of the geese: BW and ADG increased by 3.67% and 6.94%, respectively (*p* < 0.01), and the feed-to-gain ratio (F/G) decreased by 7.72% (*p* < 0.05).

### 3.2. Slaughter Performance

The slaughter performance is shown in Table 3. The abdominal and intestinal fat rates of geese in the LPR_0.44_ group were lower (*p* < 0.05) than those in the LPR_0.26_ group, while breast and leg muscle yields were higher in the LPR_0.44_ group (*p* < 0.05). The yield of carcass, semi-eviscerated carcass, eviscerated carcass, and skin and subcutaneous fat did not differ significantly (*p* > 0.05).

### 3.3. Meat Quality Assessment

Meat quality assessment (Table 4) revealed significantly higher redness values (a*) at 45 min postmortem in the breast muscles of LPR_0.34_ and LPR_0.44_ groups compared with NPR_0.34_ group (*p* < 0.05). No significant differences were observed in other meat quality indexes among groups (*p* > 0.05).

### 3.4. Nutrient Composition in Breast Muscle and Liver

As shown in Table 5, the dry matter, crude protein, and crude fat contents in the breast muscle and liver were not affected by different AM/AP ratios in low-protein diets (*p* > 0.05).

### 3.5. Muscle Fatty Acids

Thirty-seven fatty acid methyl esters (FAMEs) were measured in breast muscle by gas chromatography. As shown in Table 6, compared with the LPR_0.26_ group, the C6:0, C8:0, C11:0, C12:0, and C13:0 contents of saturated fatty acids in the breast muscle were significantly higher in the LPR_0.44_ group (*p* < 0.05), whereas the concentration of C18:0 was significantly lower (*p* < 0.05). No significant differences were found in MUFA and PUFA contents among groups (*p* > 0.05).

### 3.6. Serum Metabolites

The effects of low-protein diets with different AM/AP ratios on serum metabolites of goslings are shown in Table 7. The albumin-to-globulin ratio in the LPR_0.44_ group was higher than that in the LPR_0.26_ group (*p* < 0.05), whereas no significant differences were detected between the NPR_0.34_ and LPR_0.34_ groups (*p* > 0.05), demonstrating that protein reduction did not affect the A/G ratio. TCHO and TG levels were significantly lowered in the LPR_0.44_ group compared to the LPR_0.26_ group (*p* < 0.05). The remaining biochemical indices showed no significant variations among groups (*p* > 0.05).

### 3.7. Liver Lipid Metabolism Enzyme

The liver enzyme activity results are presented in Table 8. HL activity was significantly higher in the NPR_0.34_ and LPR_0.26_ groups (*p* < 0.01). Although no significant effects were observed on LPL, ACC, and FAS activities in the liver (*p* > 0.05), certain numerical trends were evident.

### 3.8. Liver Fatty Acids

As shown in Table 9, among the saturated fatty acids (SFAs), the contents of the short-chain fatty acids C4:0 and C12:0 were significantly reduced in the LPR_0.44_ group compared to the LPR_0.26_ group (*p* < 0.05), whereas the content of the medium-chain fatty acid C14:0 was significantly increased (*p* < 0.01). Among the monounsaturated fatty acids (MUFAs), only C24:1 showed significant group differences (*p* < 0.01), with its content being significantly higher in the LPR_0.26_ group compared to the LPR_0.34_ and LPR_0.44_ groups. Regarding polyunsaturated fatty acids (PUFAs), the content of linoleic acid (C18:2n6c) was highest in the LPR_0.26_ group and significantly decreased in the LPR_0.44_ group (*p* < 0.01). The content of docosadienoic acid (C22:2) was significantly higher in the NPR_0.34_ group than in the LPR_0.34_ group (*p* < 0.05).

### 3.9. Intestinal Digesta Enzyme

As shown in Table 10, The effect of different AM/AP ratios in low-protein diets on the activities of lipase, amylase, trypsin, and chymotrypsin was not significant in the digesta of all intestinal segments of goslings (*p* > 0.05).

### 3.10. Nutrient Digestibility

Outcomes for nutrient digestibility are shown in Table 11, the four groups showed no significant differences in all nutrient utilization indices (*p* > 0.05).

### 3.11. Intestinal Histomorphology

Table 12 shows the effect of different AM/AP ratios in low-protein diets on villus height, crypt depth, and muscularis thickness in the small intestine of goslings. No significant differences were observed among groups (*p* > 0.05).

## 4. Discussion

The current study showed that increasing the AM/AP ratios in the diet significantly enhanced the growth performance of goslings. Both BW and ADG exhibited a linear increasing trend with elevated AM/AP ratios in the diet. These findings align with previous reports [25], The reason might be caused by the slower digestion and absorption of high-amylose starch in the intestinal tract, which further provides sustained energy release and promotes animal growth [26]. The slow-digesting properties of high-amylose starch facilitate gradual glucose release in the foregut, maintaining blood glucose homeostasis and reducing energy metabolism losses. The undigested portion undergoes hindgut fermentation, producing beneficial metabolites like short-chain fatty acids that support intestinal health, improve nutrient utilization, and reduce the F/G [27]. Previous studies have shown that increasing lysine by 0.2% in low-crude-protein rations improved feed conversion [28]. In the present experiment, the LPR_0.44_ group showed significantly lower F/G compared to the LPR_0.26_ and LPR_0.34_ groups. This improvement may be attributed to the following metabolic shift: The higher AM/AP ratio in the LPR_0.44_ diet slowed starch digestion, resulting in a sustained and gradual release of glucose into the bloodstream. This stable glucose supply reduced the intestinal oxidation of amino acids for energy. Consequently, a greater proportion of amino acids passed through the intestinal mucosa into the portal vein and were transported to the liver [29]. This shift enhanced hepatic protein synthesis, thereby promoting muscle protein deposition [30], leading to increased body weight and a consequent improvement in F/G. To examine whether low protein levels affected 63-day-old gosling performance, we compared the NPR_0.34_ and LPR_0.34_ groups. This study demonstrated that reducing the dietary protein content by 2% did not affect growth performance or feed utilization, which is consistent with previous studies [5,31]. These studies demonstrated that growth performance remains unaffected by crude protein reduction when accompanied by appropriate amino acid supplementation that meets poultry’s basic requirements [24]. Therefore, the LPR_0.34_ group was shown to effectively conserve protein resources while maintaining poultry growth performance and feed utilization.

To balance ration proportions, low-protein diets typically increase starch content by raising the proportion of grains (corn/wheat) [5], This results in an excess of available energy, which is subsequently channeled into abdominal fat deposition [6]. Numerous studies have demonstrated that body fat deposition primarily depends on energy intake [32,33,34,35]. Slowly digestible starch reduces abdominal fat accumulation by decreasing glycemic and insulin responses, suppressing hepatic lipid synthesis-related gene expression, and lowering lipogenic enzyme activity [36]. Similar effects have been observed across multiple species. It has been reported that high-amylose starch reduced whole-body fat, subcutaneous fat, and visceral fat in mice without affecting total body weight [37]. Similarly, the abdominal fat percentage in broilers fed glutinous corn starch feed (low AM/AP ratio) was significantly higher than in other groups [38]. In pigs, feeds with higher amylose content improved growth performance and loin eye area while reducing backfat depth in fattening pigs [39]. In this study, increasing the AM/AP ratio in low-protein feeds significantly decreased abdominal and intestinal fat rates, indicating that this adjustment remains effective for enhancing lipid metabolism and reducing fat deposition in geese [14].

Physical parameters, including cooking loss, shear force, drip loss rate, pH, and meat color, are commonly used to evaluate meat quality. The a* value reflects meat redness, with higher values generally indicating more desirable meat characteristics [40]. The study demonstrated that low-protein, high-AM/AP ratio diets were more effective in enhancing a* at 45 min postmortem. The phenomenon was attributed to elevated MyHC I or IIa expression in muscles from low-protein diet regimens, as these fiber types contain greater myoglobin and mitochondrial content, leading to redder coloration—a key factor in increased a* values [41]. Additionally, intramuscular fat and fatty acid composition serve as important indicators of meat tenderness and flavor. Pigs fed high-amylose diets exhibited increased intramuscular fat content, thereby improving tenderness, juiciness, and flavor [20]. However, in this experiment, low-protein diets with varying AM/AP ratios did not significantly affect breast or leg muscle fat content. This may be because intramuscular fat (IMF) content is mainly determined by a stable genetic background, and short-term nutritional intervention is insufficient to alter genetic traits [42]. Moreover, dietary energy level is also a key factor regulating IMF deposition in geese, with a clear threshold effect. In Xupu geese, a significant increase in breast muscle fat content was observed only when the dietary energy reached 12.55 MJ/kg, compared to the control group at 11.70 MJ/kg [43]. The energy level used in our trial (11.20 MJ/kg) was below this effective threshold, falling within a relatively insensitive range for IMF deposition in response to energy variation, which explains the absence of significant changes. Intramuscular fatty acid composition determines nutritional value and oxidative stability and is a crucial lipid component [44]. Saturated fatty acids, with their stable chemical structure, tend to accumulate, whereas excessive unsaturated fatty acids may undergo oxidation, compromising meat flavor [45]. This study indicated that low-protein, high-AM/AP ratio diets altered gosling muscle fatty acid composition by increasing saturated fatty acid content without significantly affecting unsaturated fatty acids, suggesting that these formulations help maintain optimal polyunsaturated/saturated fatty acid ratios to preserve meat quality and nutritional value.

Serum levels of glucose (GLU), triglyceride (TG), total cholesterol (TC), high-density lipoprotein (HDL), and low-density lipoprotein (LDL) are widely used to reflect the body’s carbohydrate and lipid metabolism [46]. In this experiment, low-protein, high-amylose feed reduced serum TG and TC levels. This is consistent with previous research showing that resistant starch complexes significantly decrease serum TG and TC levels while effectively preventing excessive fat deposition through improved lipid metabolism [9,47]. These findings suggest that under conditions of a high AM/AP ratio, lipid metabolism may be regulated through AMPK activation, which enhances fatty acid oxidation [48], and through the suppression of fatty acid synthesis due to reduced insulin levels, thereby further lowering serum TC and TG levels [49]. Additionally, the reduced insulin levels may further inhibit cholesterol synthesis, contributing to improved lipid profiles [50]. This dual regulation of lipid metabolism under high AM/AP ratio conditions may improve lipid and cholesterol levels in goslings [51].

Fat in the liver is primarily converted from dietary carbohydrates and energy. Fatty acid synthase (FAS) is the primary enzyme responsible for hepatic fatty acid synthesis that regulates hepatic lipogenesis by catalyzing acetyl-CoA carboxylase (ACC) to generate malonyl-CoA, which in turn converts glucose to palmitic acid and ultimately facilitates the synthesis of fatty acids and triglycerides (TG) [52,53]. Hepatic lipase (HL) catalyzes the hydrolysis of triglycerides and phospholipids, participating in the conversion of VLDL to LDL and the remodeling of plasma lipoproteins [54]. Studies have reported that increasing the proportion of amylose in feed can reduce fat deposition by inhibiting the activity of key liposynthetic enzymes [55]. The main reason is that amylose regulates blood sugar levels in the body by slowly releasing glucose and inhibits fatty acid synthase activity, thereby ultimately reducing fat deposition [11]. Furthermore, it has been shown that lotus seed resistant starch reduces serum TG levels by promoting the oxidation and catabolism of fatty acids, ultimately inhibiting fat formation [9,56]. This study indicated that a high AM/AP ratio diet significantly reduced hepatic production of SFAs such as C4:0, C12:0, and C24:1 as well as PUFAs such as C18:2n6c by decreasing hepatic enzyme activities of FAS and HL. The decrease in hepatic lipase (HL) activity has a specific impact on lipid metabolism by impairing the conversion of very low-density lipoproteins (VLDLs) to low-density lipoproteins (LDLs), which is a critical step in lipoprotein metabolism [57]. HL, as a key lipolytic enzyme, regulates plasma triglyceride (TG) levels by hydrolyzing triglycerides in lipoproteins [54]. Reduced HL activity leads to delayed clearance of circulating lipoproteins, causing an accumulation of triglycerides in the bloodstream. This disruption in lipoprotein metabolism alters the metabolic signals sent to the liver [58], potentially triggering a change in the expression of lipogenic enzymes such as fatty acid synthase (FAS). Consequently, this may inhibit hepatic fatty acid synthesis, particularly reducing the production of certain fatty acids like C4:0 and C12:0. Thus, the decrease in HL activity affects both lipoprotein conversion and fatty acid synthesis inhibition, ultimately influencing overall lipid homeostasis. FAS is mainly responsible for the de novo synthesis of saturated fatty acids (SFAs) and MUFAs. Although there was no significant change in the activities of ACC and FAS in this study, a numerically decreasing trend was observed., which could lead to the reduction in the specific fatty acid products it is responsible for synthesizing (such as C4:0, C12:0). This down-regulation trend could be associated with the observed reduction in lipid deposition and cholesterol content, suggesting a potential mechanism for mitigating fat accumulation [59].

The ability of poultry to digest and absorb nutrients is typically expressed as the apparent digestibility of the nutrients, which is an important indicator of dietary ingredient value. The capacity of an animal to utilize feed nutrients is often evaluated by measuring the activities of individual intestinal digestive enzymes. It has been found that dietary protein levels within a certain range have no significant effect on digestive performance [18]. The results of the present study are consistent with these findings, showing no significant effect on intestinal enzyme activity or nutrient utilization between the 14% low-protein diet and the normal-protein diet. Regarding starch structure, the complex molecular structure of high amylose limited the exposure of α-glycosidic bonds to digestive enzymes and prolongs its gastrointestinal transit time, which resulted in it being less susceptible to degradation by α-amylase compared to amylopectin [60]. Research has further demonstrated that an increase in the dietary AM/AP ratio leads to a significant decrease in α-amylase activity, thereby reducing apparent energy and starch digestibility at the terminal ileum [12,61,62]. However, in the present study, different AM/AP ratios were observed to have no significant effect on the activities of intestinal α-amylase and other related enzymes. This discrepancy might be attributed to the small differences between groups and the short 28-day experimental period [12]. While we considered the impact of AM/AP ratios and experimental duration, the observed effects on lipid metabolism are more likely due to total starch content rather than starch structure [5,63]. Given the low sensitivity of intestinal amylase to starch structure in goslings, the AM/AP ratio appears to have minimal physiological impact in this context. Since intestinal enzyme activity is closely related to nutrient utilization, the absence of significant differences in nutrient digestibility among the treatment groups indicates that the digestive capacity of the goslings remained stable across all dietary treatments.

Intestinal villus height, width, and number serve as key indicators of intestinal digestion and absorption. Increased villus height (VH) and VH-to-crypt depth (VH/CD) ratio are typically associated with greater absorptive surface area and enhanced absorption efficiency. Taller villi increase intestinal mucosal surface area, improving nutrient contact and absorption, whereas shallower crypts indicate higher intestinal epithelial cell maturation rates and stronger absorptive function [64]. The results of this experiment showed no significant differences in intestinal morphological parameters among treatment groups, It was indicated that within the AM/AP ratio and period set in this study, dietary treatment did not cause detectable negative impacts on intestinal health; this was manifested as an intact intestinal barrier and stable digestive function. This finding has important practical significance because it shows that adjusting the diet within this AM/AP ratio range will not damage the intestinal health foundation of geese, providing a key basis for the safe application of low-protein diets. It was also demonstrated by relevant research that the villus height, crypt depth, and the villus height-to-crypt depth ratio in the ileum were not affected by different starch ratios [18]. However, other studies have reported that higher proportions of amylose or resistant starch could lead to alterations in intestinal morphology [65,66]. The differences in these results may arise from differences in experimental design among different studies, including experimental poultry species, intestinal segments observed, different levels of starch addition and experimental durations.

Nevertheless, this study has certain limitations. First, the 28-day trial was confined to a single growth phase, which constrains our understanding of how the AM/AP ratio functions across different physiological stages. Second, by not examining the gut microbiota, we cannot clarify its potential role in mediating the observed effects on lipid metabolism and intestinal function. Finally, the long-term implications of modulating the AM/AP ratio for intestinal health and production performance remain unclear and require validation in extended trials. Consequently, future research should encompass longer durations and multiple growth phases, integrated with systematic gut microbiome analysis. This comprehensive approach is essential to fully elucidate the mechanisms through which different AM/AP ratios influence growth performance, intestinal health, and lipid metabolism in poultry.

## 5. Conclusions

The present experiment found that with higher AM/AP ratios at low protein, the ADG of goslings increased significantly and F/G decreased significantly, while muscle yield and quality increased significantly. When the AM/AP ratio was 0.44, lipid metabolism was effectively regulated (by lowering blood lipid cholesterol content and controlling abdominal and intestinal lipid deposition) through inhibition of hepatic fatty acid synthase activity and reduction in SFA and MUFA production. Additionally, the low-protein feed with an AM/AP ratio of 0.44 (LPR_0.44_) showed no adverse effects on gut health or nutrient utilization. Based on these findings, it is recommended to appropriately increase the AM/AP ratio in low-protein diets for goslings to improve their growth performance and lipid metabolism.

## Figures and Tables

**Table 1:** Feed composition and nutritional levels for 35–63 day old goslings (dry matter basis) (%).

Items	NPR_0.34_	LPR_0.26_	LPR_0.34_	LPR_0.44_
Corn	61.85	55.20	65.30	60.78
Soybean meal	23.60	15.80	17.44	18.00
High-amylose corn starch	0.00	0.00	0.00	4.00
High-amylopectin corn starch	0.00	8.50	0.00	0.00
Wheat bran	5.64	12.18	7.92	8.15
Rice hull	5.29	4.50	5.48	5.30
Limestone	1.05	1.08	1.08	1.05
CaHPO_4_	1.11	1.10	1.12	1.10
DL-methionine	0.16	0.19	0.20	0.19
Lysine	0.00	0.15	0.16	0.13
Salt	0.30	0.30	0.30	0.30
Premix ^1^	1.00	1.00	1.00	1.00
Total	100.00	100.00	100.00	100.00
Nutrient levels, % ^2^				
Metabolizable Energy (MJ/kg)	11.22	11.21	11.20	11.18
Crude protein	16.02	14.01	14.06	14.00
Crude fiber	5.47	5.06	5.75	5.30
Lysine	0.83	0.82	0.83	0.82
Methionine	0.43	0.43	0.43	0.43
Calcium	0.84	0.79	0.86	0.80
Total phosphorus	0.63	0.62	0.62	0.62
Amylose	10.30	9.19	10.87	12.94
Amylopectin	30.18	35.27	31.87	29.17
AM/AP ratio	0.34	0.26	0.34	0.43

^1^ The premix contains the following per kilogram: VA, 9000 IU; VD_3_, 3000 IU; VE, 25 mg; VK_3_, 1.5 mg; VB_1_, 0.9 mg; VB_2_, 8 mg; VB_6_, 3.2 mg; VB_12_, 0.01 mg; nicotinic acid, 45 mg; pantothenic acid, 11 mg; folic acid, 0.65 mg; choline chloride, 350 mg; biotin, 0.05 mg; Fe, 60 mg; Cu, 10 mg; Mn, 95 mg; Zn, 90 mg; I, 0.5 mg; Se, 0.3 mg. ^2^ Analytically measured parameters included crude protein, crude fiber, calcium, and total phosphorus, with the remaining values being calculated.

**Table 2:** Effects of different dietary treatments on the growth performance of goslings 35–63 days of age.

Items	NPR_0.34_	LPR_0.26_	LPR_0.34_	LPR_0.44_	SEM	*p*-Value
BW (kg)	3.79 ^b^	3.81 ^b^	3.87 ^ab^	3.95 ^a^	0.020	0.007
ADG (g)	59.52 ^c^	60.54 ^bc^	62.30 ^ab^	64.74 ^a^	0.518	<0.001
ADFI (g)	284.99	289.51	284.86	286.13	1.067	0.397
F/G	4.79 ^a^	4.79 ^a^	4.58 ^ab^	4.42 ^b^	0.043	0.001

BW: body weight, ADFI: average daily feed intake, ADG: average daily gain, F/G: feed-to-gain ratio. (a,b,c) Means with different superscripts within the same row differ significantly (*p* < 0.05).

**Table 3:** Effects of different dietary treatments on slaughter performance of 63-day-old goslings (%).

Items	NPR_0.34_	LPR_0.26_	LPR_0.34_	LPR_0.44_	SEM	*p*-Value
Carcass yield	88.41	88.51	89.13	89.18	0.221	0.503
Semi-eviscerated carcass yield	81.54	81.74	82.43	81.25	0.240	0.350
Eviscerated carcass yield	73.29	72.42	73.70	74.28	0.289	0.131
Breast muscle yield	9.72 ^ab^	9.29 ^b^	9.33 ^b^	10.61 ^a^	0.166	0.010
Leg muscle yield	12.94 ^ab^	12.4 ^b^	13.41 ^ab^	13.85 ^a^	0.185	0.046
Abdominal fat yield	3.47 ^ab^	3.70 ^a^	3.24 ^ab^	3.00 ^b^	0.096	0.038
Skin and subcutaneous fat yield	21.76	23.68	21.78	22.03	0.318	0.114
Mesenteric fat yield	2.42 ^bc^	3.02 ^a^	2.86 ^ab^	2.30 ^c^	0.113	0.040

(a,b,c) Means with different superscripts within the same row differ significantly (*p* < 0.05).

**Table 4:** Effects of different dietary treatments on meat quality of 63-day-old goslings.

Items	Cooking Loss, %	Shearing Strength, N	Drip Loss, %	pH_45min_	pH_24h_	Color_45min_		
						L* ^1^	a* ^1^	b* ^1^
NPR_0.34_	30.05	57.45	3.85	6.57	6.15	33.63	15.06 ^b^	6.53
LPR_0.26_	29.14	47.49	4.16	6.35	6.11	34.26	16.57 ^ab^	7.40
LPR_0.34_	29.03	60.61	4.02	6.26	6.03	30.84	17.89 ^a^	9.20
LPR_0.44_	29.05	57.89	4.24	6.40	6.07	32.42	17.61 ^a^	9.00
SEM	0.667	1.861	0.414	0.058	0.018	0.851	0.409	0.428
*p*-Value	0.948	0.054	0.307	0.322	0.108	0.531	0.048	0.068

^1^ L*: brightness, a*: redness, b*: yellowness. (a,b) Means with different superscripts within the same row differ significantly (*p* < 0.05).

**Table 5:** Effects of different dietary treatments on routine nutrients composition in breast muscle and liver of 63-day-old goslings (%).

Items	Breast Muscle			Liver		
	Dry Matter	Crude Protein	Crude Fat	Dry Matter	Crude Protein	Crude Fat
NPR_0.34_	26.37	17.61	2.60	33.66	17.83	3.12
LPR_0.26_	26.24	16.75	2.82	34.35	17.23	3.38
LPR_0.34_	26.07	17.08	2.93	35.92	16.54	3.71
LPR_0.44_	25.83	17.16	2.95	36.11	17.07	3.53
SEM	0.220	0.210	0.090	0.452	0.198	0.178
*p*-Value	0.855	0.605	0.501	0.166	0.213	0.719

**Table 6:** Effects of different dietary treatments on breast muscle fatty acid composition of 63-day-old goslings (g/100 g).

Items	NPR_0.34_	LPR_0.26_	LPR_0.34_	LPR_0.44_	SEM	*p*-Value
SFA						
C4:0	13.07	11.94	12.60	15.07	0.49	0.234
C6:0	1.61 ^b^	1.28 ^b^	2.12 ^ab^	3.26 ^a^	0.26	0.012
C8:0	0.68 ^b^	0.58 ^b^	0.76 ^b^	1.40 ^a^	0.10	0.004
C10:0	2.08	1.81	2.19	2.90	0.17	0.114
C11:0	0.07 ^b^	0.07 ^b^	0.08 ^b^	0.14 ^a^	0.01	0.004
C12:0	2.88 ^b^	3.98 ^b^	6.94 ^a^	8.64 ^a^	0.63	<0.000
C13:0	3.51 ^b^	3.81 ^b^	5.24 ^b^	8.24 ^a^	0.47	<0.000
C14:0	10.72	10.84	16.76	14.11	1.31	0.326
C15:1	0.69	0.62	0.81	1.08	0.07	0.101
C16:0	10.06	9.13	8.92	18.6	1.64	0.157
C17:0	15.30	13.46	15.24	19.71	1.13	0.253
C18:0	20.61 ^a^	22.31 ^a^	22.69 ^a^	9.84 ^b^	1.57	0.014
C20:0	15.77	8.56	14.43	9.94	1.92	0.533
C21:0	10.33	8.34	10.15	16.47	1.40	0.182
C22:0	20.84	26.72	20.26	37.94	3.21	0.246
C23:0	7.12	7.32	8.10	13.56	1.00	0.069
MUFA						
C24:1	0.07	0.07	0.10	0.27	0.03	0.066
C14:1	19.70	11.63	9.80	8.46	2.524	0.455
C15:0	6.03	3.63	3.67	5.51	0.642	0.457
C16:1	8.99	7.70	8.38	7.87	0.870	0.963
C17:1	0.69	0.62	0.81	1.08	0.258	0.343
C18:1n9t	0.07 ^b^	0.07 ^b^	0.08 ^ab^	0.13 ^a^	0.009	0.016
C18:1n9c	26.68	22.47	30.76	37.61	2.661	0.236
C20:3n6	0.07 ^b^	0.07 ^b^	0.06 ^b^	0.15 ^a^	0.013	0.025
C22:1n9	11.61	19.49	19.05	19.12	2.059	0.521
C24:0	4.78	8.45	4.52	7.37	1.060	0.515
PUFA						
C18:2n6t	0.50	0.39	0.46	0.41	0.042	0.824
C18:2n6c	13.41	11.15	13.19	9.71	1.827	0.891
C18:3n6	7.19	5.51	6.11	7.39	1.156	0.939
C20:1	5.54	3.63	4.32	6.44	0.536	0.311
C18:3n3	6.03	4.46	5.76	8.93	0.710	0.124
C20:2	2.93	5.31	5.33	4.27	0.642	0.551
C20:3n3	7.12	7.32	8.10	13.56	0.999	0.069
C20:4n6	0.13	0.11	0.16	0.21	0.021	0.364
C22:2	12.85	10.13	12.70	8.42	0.783	0.124
C20:5	25.52	16.56	15.04	8.00	3.149	0.310
C22:6n3	0.37	0.16	0.28	0.34	0.054	0.582

(a,b) Means with different superscripts within the same row differ significantly (*p* < 0.05).

**Table 7:** Effects of different dietary treatments on Serum metabolites of 63-day-old goslings.

Items	TP g/L	ALB g/L	GLB g/L	A/G	GLU mmol/L	TCHO mmol/L	TG mmol/L	HDL mmol/L	LDL mmol/L	UREA mmol/L	CREA μmol/L
NPR_0.34_	39.72	13.58	26.13	0.52 ^ab^	10.39	4.13 ^ab^	2.05 ^ab^	2.44	2.41	16.83	0.78
LPR_0.26_	43.08	13.60	29.48	0.50 ^b^	10.95	4.55 ^a^	2.51 ^a^	2.12	2.19	21.00	0.73
LPR_0.34_	40.63	14.40	26.23	0.55 ^ab^	10.85	4.31 ^ab^	2.01 ^ab^	2.49	2.64	18.00	0.70
LPR_0.44_	40.85	15.20	25.63	0.59 ^a^	10.33	3.88 ^b^	1.38 ^b^	2.29	2.47	25.17	0.75
SEM	0.723	0.300	0.658	0.012	0.157	0.087	0.145	0.057	0.064	2.195	0.028
*p*-Value	0.426	0.174	0.142	0.034	0.406	0.036	0.038	0.082	0.082	0.570	0.792

Abbreviations: TP, total protein; ALB, albumin; GLB, globulin; A/G, Albumin-to-Globulin Ratio; GLU, glucose; TCHO, total cholesterol; TG, triglycerides; HDL, high-density lipoprotein; LDL, low-density lipoprotein; UREA, urea nitrogen; CREA, creatinine. (a,b) Means with different superscripts within the same row differ significantly (*p* < 0.05).

**Table 8:** Effects of different dietary treatments on the activity of liver lipid metabolism enzyme in 63-day-old goslings (U/L).

Items	ACC	FAS	LPL	HL
NPR_0.34_	10.28	301.83	135.20	35.22 ^a^
LPR_0.26_	11.24	477.67	113.73	35.14 ^a^
LPR_0.34_	10.03	412.85	101.66	29.68 ^b^
LPR_0.44_	9.79	419.06	120.24	29.45 ^b^
SEM	0.407	27.582	5.380	0.943
*p*-Value	0.637	0.125	0.165	0.019

Abbreviations: ACC: acetyl-coA carboxylase; FAS: fatty acid synthase; LPL: lipoprotein lipase; HL: hepatic lipase. (a,b) Means with different superscripts within the same row differ significantly (*p* < 0.05).

**Table 9:** Effects of different dietary treatments on the fatty acid composition of liver in 63-day-old goslings (g/100 g).

Items	NPR_0.34_	LPR_0.26_	LPR_0.34_	LPR_0.44_	SEM	*p*-Value
SFA						
C4:0	1.3472 ^b^	1.7024 ^a^	1.7287 ^a^	1.3919 ^b^	0.063	0.027
C6:0	0.0504	0.0255	0.0534	0.0802	0.011	0.562
C8:0	0.3792	0.3079	0.3057	0.3231	0.045	0.951
C10:0	0.0033	0.0037	0.0057	0.0054	0.000	0.184
C11:0	0.0638	0.0496	0.0553	0.0486	0.004	0.664
C12:0	0.1772 ^b^	0.5412 ^a^	0.1888 ^b^	0.2240 ^b^	0.036	<0.001
C13:0	0.0364	0.0426	0.0309	0.0286	0.003	0.448
C14:0	0.3287 ^b^	0.2178 ^b^	0.4288 ^ab^	0.6241 ^a^	0.049	0.002
C15:1	0.0206	0.0143	0.0389	0.0185	0.004	0.176
C16:0	0.3092	0.3872	0.5441	0.2902	0.049	0.255
C17:0	0.0041	0.0031	0.0036	0.0035	0.000	0.829
C18:0	0.0062	0.0059	0.0047	0.0044	0.001	0.684
C20:0	0.0069	0.0074	0.0068	0.0073	0.000	0.948
C21:0	0.0075	0.0081	0.0084	0.0057	0.001	0.894
C22:0	0.0027	0.0023	0.0038	0.0042	0.001	0.785
C23:0	0.0028	0.0021	0.0015	0.0029	0.000	0.475
MUFA						
C24:1	0.0054 ^a^	0.0057 ^a^	0.0024 ^b^	0.0022 ^b^	0.000	0.001
C14:1	0.0897	0.0666	0.1056	0.1862	0.027	0.423
C15:0	0.0029	0.0036	0.0036	0.0034	0.000	0.902
C16:1	0.1103	0.1365	0.2091	0.1374	0.025	0.553
C17:1	0.0077	0.0058	0.0065	0.0068	0.001	0.915
C18:1n9t	0.0046	0.0044	0.0059	0.0035	0.001	0.475
C18:1n9c	0.0067	0.0089	0.0024	0.0022	0.001	0.147
C20:3n6	0.0020	0.0057	0.0097	0.0065	0.001	0.175
C22:1n9	0.0033	0.0045	0.0057	0.0037	0.000	0.322
C24:0	0.0069	0.0055	0.0083	0.0056	0.001	0.485
PUFA						
C18:2n6t	0.0043	0.0043	0.0045	0.0035	0.000	0.406
C18:2n6c	0.0044 ^ab^	0.0070 ^a^	0.0044 ^ab^	0.0021 ^b^	0.001	0.008
C18:3n6	0.0018	0.0024	0.0026	0.0012	0.000	0.437
C20:1	0.0051	0.0055	0.0057	0.0046	0.001	0.935
C18:3n3	0.0030	0.009	0.0057	0.0058	0.001	0.252
C20:2	0.0013	0.0013	0.0014	0.0021	0.000	0.120
C20:3n3	0.0039	0.0054	0.0042	0.0049	0.000	0.673
C20:4n6	0.0079	0.0055	0.0065	0.0052	0.001	0.636
C22:2	0.0068 ^a^	0.0060 ^ab^	0.0024 ^b^	0.0035 ^ab^	0.001	0.030
C20:5	0.0046	0.0038	0.0054	0.0036	0.000	0.395
C22:6n3	0.0041	0.0034	0.0023	0.0037	0.001	0.809

(a,b) Means with different superscripts within the same row differ significantly (*p* < 0.05).

**Table 10:** Effects of different dietary treatments on the activities of digestive enzymes in the Intestinal chyme of 63-day-old goslings.

Items	Intestinal Segment	NPR_0.34_	LPR_0.26_	LPR_0.34_	LPR_0.44_	SEM	*p*-Value
Lipase (U/gprot)	Duodenum	61.35	60.60	57.61	54.40	3.912	0.942
	Jejunum	44.32	47.17	44.97	45.88	2.450	0.981
	Ileum	50.56	47.97	42.91	47.99	5.239	0.972
α-amylase (U/mgprot)	Duodenum	5.38	6.97	4.39	6.27	0.727	0.656
	Jejunum	9.19	9.34	7.79	9.90	1.330	0.964
	Ileum	13.35	13.42	15.37	15.73	2.296	0.601
Trypsin (U/mgprot)	Duodenum	955.85	1022.32	962.18	957.77	33.367	0.891
	Jejunum	882.10	824.85	798.16	884.10	16.251	0.155
	Ileum	639.68	623.36	740.13	776.77	36.270	0.415
Chymotrypsin (U/mgprot)	Duodenum	2.58	2.80	2.38	2.54	0.115	0.671
	Jejunum	2.09	2.02	2.12	2.01	0.199	0.998
	Ileum	2.98	2.83	3.01	2.75	0.271	0.989

**Table 11:** Effects of different dietary treatments on nutrient digestibility in goslings from 63 d (%).

Items	Energy	Dry Matter	Crude Ash	Crude Protein	Crude Fat	Crude Fiber	Calcium	Phosphorus
NPR_0.34_	78.26	72.12	44.55	47.99	53.22	24.65	55.09	56.97
LPR_0.26_	78.37	71.57	52.46	40.17	56.45	21.39	59.57	62.87
LPR_0.34_	76.38	70.36	45.19	42.58	52.62	23.06	54.03	59.95
LPR_0.44_	78.03	69.35	42.99	45.11	54.23	26.84	54.52	63.92
SEM	0.503	0.564	1.729	2.347	1.126	2.236	1.754	1.245
*p*-Value	0.427	0.369	0.262	0.731	0.613	0.868	0.696	0.260

**Table 12:** Effects of different dietary treatments on the morphology of Intestinal tissues in 63-day-old goslings.

Items	Intestinal Segment	NPR_0.34_	LPR_0.26_	LPR_0.34_	LPR_0.44_	SEM	*p*-Value
Villus height (μm)	Duodenum	1098.67	1154.83	1054.67	1081.75	26.109	0.624
	Jejunum	1301.88	1362.29	1300.83	1356.17	30.487	0.855
	Ileum	1114.79	1124.71	1156.00	1096.92	14.046	0.550
Crypt depth (μm)	Duodenum	274.92	252.33	263.00	307.33	11.845	0.421
	Jejunum	262.67	285.67	249.50	282.00	6.367	0.153
	Ileum	225.75	208.00	207.00	217.44	3.308	0.153
Muscle layer thickness (μm)	Duodenum	394.67	330.56	342.56	324.22	10.974	0.062
	Jejunum	400.00	355.33	329.44	389.11	11.788	0.111
	Ileum	377.50	358.17	320.83	324.22	10.381	0.146
V/C ^1^	Duodenum	3.55	4.31	4.05	3.59	0.14	0.174
	Jejunum	3.99	4.13	4.29	4.65	0.19	0.697
	Ileum	4.64	4.73	5.34	5.01	0.35	0.910

^1^ V/C, villus height/crypt depth.

## Data Availability

The original contributions presented in this study are included in the article. Further inquiries can be directed to the corresponding author.
